# Case Report: Noninvasive Clinical Intervention of REBACIN® on Histologic Regression of High Grade Cervical Intraepithelial Neoplasia

**DOI:** 10.3389/fmed.2021.627355

**Published:** 2021-07-20

**Authors:** Fan Wang, Rong Liu, Yan Ma, Dai-Fei Wu, Liu-Hong Deng, Sheng Wang, Gui-Yu Wang, Chun-Fa Zhang, Quan-Xin Qu

**Affiliations:** ^1^Department of Obstetrics and Gynaecology, Tianjin First Central Hospital, Tianjin, China; ^2^Department of Molecular Virology, SR Life Sciences Institute, Clarksburg, MD, United States; ^3^Division of Medical Biology, Key Laboratory of Protein Engineering and Drug Development of Hainan, Haikou, China; ^4^Taizhou Antiviral Medical Research and Development Center, Taizhou, China

**Keywords:** Human papilloma virus (HPV), cervical cancer, regression, CIN2, REBACIN^®^, E6/E7

## Abstract

High-risk human papillomavirus (hrHPV) persistent infection is the major cause of cervical cancer. Clinical intervention of hrHPV-associated high-grade squamous intraepithelial lesion (HSIL) is critical to prevent cervical cancer, and current treatment is surgery (an invasive therapy). However, some patients refuse to do so for an afraid of potential adverse effects on future fertility or other concerns which creates a critical need for development of non-invasive therapeutic strategies. Here, we report for the first time the cases of non-invasive intervention with REBACIN®, a proprietary antiviral biologics, in clinical treatment of HSIL. From 12,958 visiting patients assessed for eligibility, 18 HSIL-patients with cervical intraepithelial neoplasia-grade 2, positive of both diffused overexpression of p16 and high-risk HPV were enrolled in this non-invasive clinical intervention mainly due to concerns of future fertility. REBACIN® was administered intravaginally every other day for 3 months (one-course) except during menstrual period, and were followed up for 6-36 months for the examination of high-risk HPV DNA, cervical cytology, and histopathology. After one to three course treatments, most cases (16/18) displayed both the regression from HSIL (CIN2) to normal cervical cytology and clearance of high-risk HPV infection. Further studies demonstrated REBACIN® significantly suppressed HPV16 E7 oncoprotein expression in a human cervical cancer cell line, which is consistent with previous finding that REBACIN® inhibits the growth of tumors induced by expression of E6/E7 oncogenes of either HPV16 or HPV18. This report indicates REBACIN® as a novel effective non-invasive clinical intervention for HSIL-patients as well for high-risk HPV persistent infection, providing a new clinical option for the non-invasive treatment of hrHPV-associated high-grade squamous intraepithelial lesion, which is worthy of further research on clinical validation and application.

## Introduction

Cervical cancer was the fourth most common cancer in women of the world and was even the first most common cancer in 42 low-resource countries in women. According to the global cancer statistics in 2018, ~570,000 cases of cervical cancer and 310,000 deaths from the disease occurred (1). It's well known that high-risk human papillomavirus (hrHPV) persistent infection is the main cause of cervical intraepithelial neoplasia (CIN) and cervical cancer (2, 3), and the preventative HPV vaccines do not able to induce strong therapeutic effects against existing HPV infections and established lesions (4, 5). Although the application of cervical cancer screening and standardized treatment of abnormal screening results effectively in reducing the incidence of cervical cancer (6), high-grade squamous intraepithelial lesion (HSIL) cases are still remained large amount in clinic. If left improperly treated, the patients should be at high risk for developing a cervical cancer.

The squamous intraepithelial lesions can be divided into low grade squamous intraepithelial lesions (LSIL) and high grade squamous intraepithelial lesions (HSIL) (7). In histopathologic diagnosis, CIN1 is classified as LSIL while CIN3 as HSIL. For CIN2, p16 immunostaining is recommended to clarify a diagnosis. If a CIN2 specimen is p16-positive, it will be classified as HSIL otherwise as LSIL (8). The standard treatment of cervical HSIL is surgery, especially for patients with local cervical lesions (9). However, this treatment may bring potential adverse effects on the future fertility of patients, and thus some patients refuse this invasive treatment, creating a critical need for the development of non-invasive therapeutic strategies for HSIL patients. In addition, since HSIL is caused by high-risk HPV persistent infection, the HSIL recurrence is still possible if high-risk HPV is not cleared completely, even though the pathological tissue is removed by surgery.

REBACIN®, a proprietary antiviral biologics, was previously reported to have a potent efficacy in the clearance of high-risk HPV persistent infections (10). We reported here the first use of a non-invasive intervention of REBACIN® in HSIL-patients. In our cases, REBACIN® treatment led to cervical lesion regression of HSIL while eliminating the high-risk HPV infection.

## Case Description

From original 12,958 visiting patients assessed for eligibility, 205 patients with high-risk HPV infection were diagnosed as CIN2 with colposcopy and cervical biopsy histopathology, and diffuse positive expression of p16 with immunohistochemistry (for details, see Supplementary Figure 1 and Table 1). According to the ASCCP rule in 2012 (9), 187 patients of them were diverted for surgical treatment while 18 patients of them refused the invasive treatment and requested for non-invasive treatment mainly due to concerns of future fertility. These 18 patients were thus enrolled in this clinical observation. Written informed consent was obtained from all patients prior to enrollment in the project.

REBACIN® was administered intravaginally every other day (0.5 g compounding agent per dose) for 3 months (one course), except during the menstrual period. After 2 months of drug withdrawal in each clinical course, the patients were followed up. HPV-DNA test, cervical erosion, thinprep cytologic test, colposcopy and histopathological examination of cervix were performed; at the same time, physical examinations including blood routine test, liver function test (LFT), and renal function test (RFT) were also performed to evaluate potential adverse reaction. All patients were followed up for 6-36 months. After each clinical course, patients still with positive of high-risk HPV and/or squamous intraepithelial lesion may accept the treatment for the second or third round.

**Figure 1:**
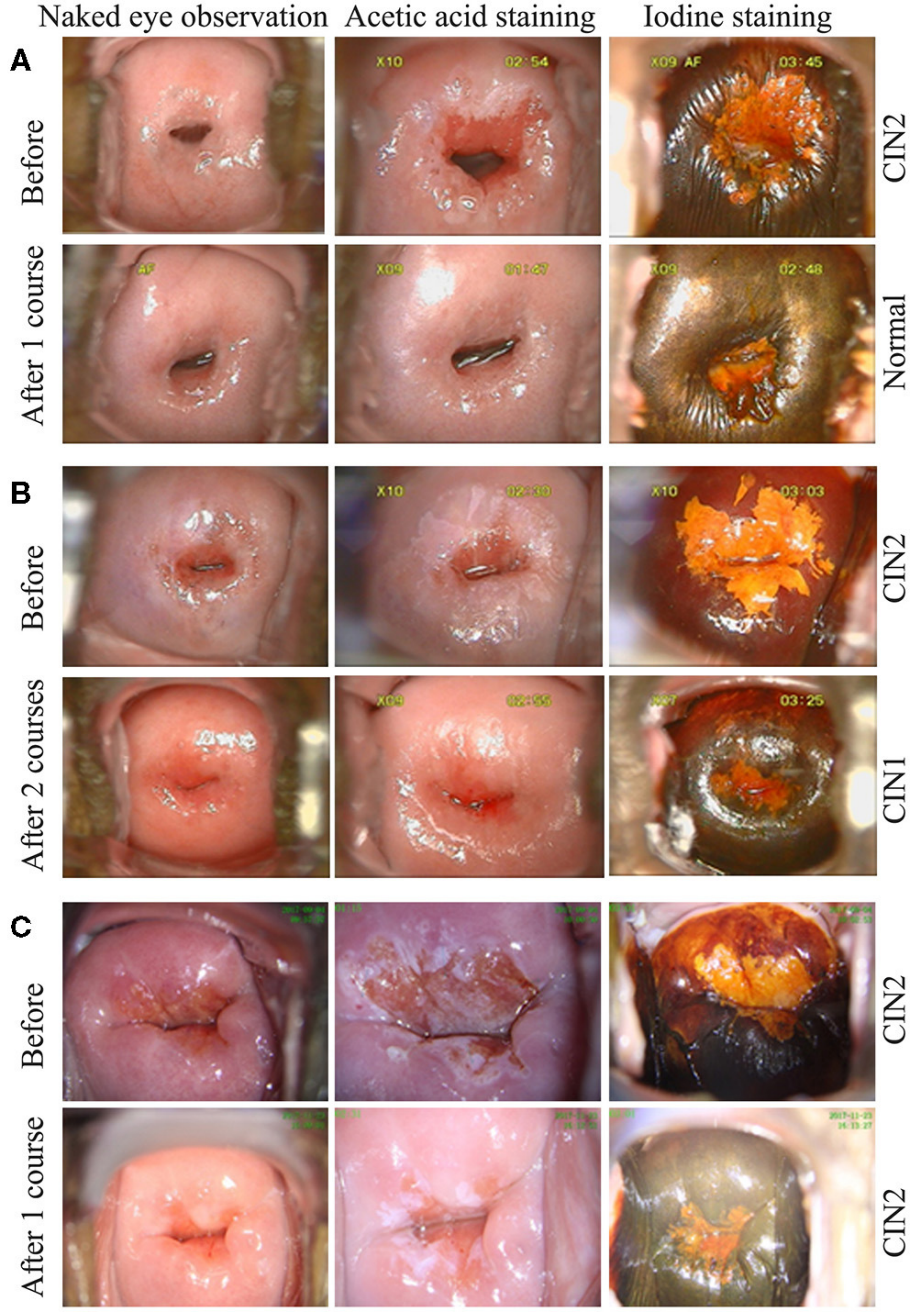
Observation *via* colposcopy before and after REBACIN® treatment. After one **(A, C)** or two **(B)** courses of REBACIN® treatment, the examination of TCT and cervical histopathology showed normal TCT **(A)**, CIN1 **(B)** and CIN2 **(C)**, respectively in lower panels. Upper panels in **(A-C)** showed the examination before REBACIN® treatment; representative pictures were shown.

**Figure 2:**
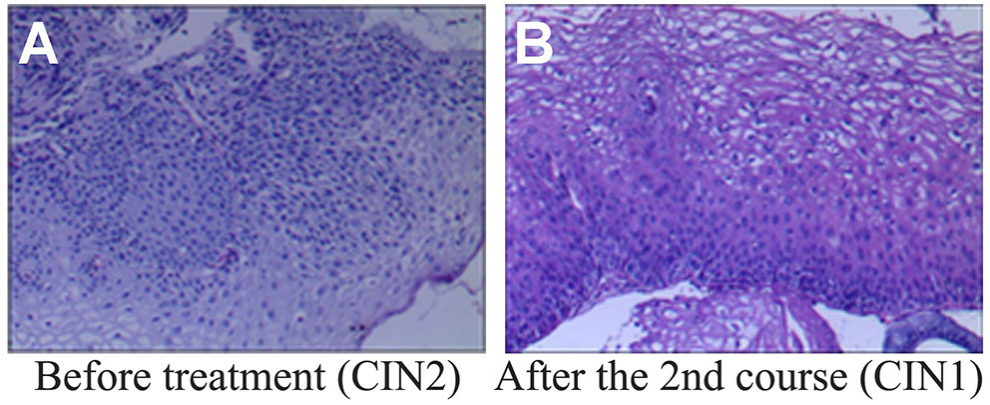
Histopathological examination before and after REBACIN® treatment. **(A)** Before REBACIN® treatment, the patient was diagnosed as CIN2 *via* the histopathology of cervical biopsy, showing a moderate atypical hyperplasia. The cells including obvious atypia cells were arranged disorderedly; the abnormally proliferating cells occupied 2/3 of the subepithelial layer. **(B)** After two courses of REBACIN® treatment, the patient was diagnosed as CIN1, showing a mild atypical hyperplasia. The cells including some atypia cells were arranged irregularly, but still maintained polarity. The abnormally proliferating cells are limited to the lower 1/3 of the subepithelial layer.

After one course (3 months) of REBACIN® treatment, 13 patients (72.22%) displayed both regression of hrHPV-associated cervical intraepithelial neoplasia from CIN2 to normal cervical cytology and clearance of high-risk HPV infection (Table 1). For the other cases: the 3rd patient was diagnosed as CIN1 from CIN2 with colposcopy and cervical biopsy, and therefore accepted the second course treatment, resulting in normal cervical cytology 2 months after the drug withdrawal, and there was no recurrence during her follow-up for up to 10 months; similarly, the 4th patient also accepted the second course treatment, and then displayed normal cervical cytology. There was no recurrence for the 4th patient during her follow-up for up to 12 months after the regression; the 6th patient displayed normal cervical cytology (Figure 1A), but showed chronic inflammation of the cervix *via* cervical biopsy. Due to strong requirement of childbirth, the 6th patient gave up the treatment of hrHPV58 infection and was administered only for follow-up observation after the first course; the 18th patient was diagnosed as CIN1 from CIN2 with colposcopy and cervical biopsy, but showed normal cervical cytology. After additional second course treatment, there was no improvement according to colposcopy and cervical biopsy (Figures 1B, 2). Thus, a third course of REBACIN® was carried out, and then showed normal cervical cytology after the treatment. So far, there was no recurrence during six-month follow-up; the 13th patient was still diagnosed as LSIL, and also CIN2 according to cervical biopsy (Figure 1C). After that, the 13th patient required surgery. Therefore, a loop electrosurgical excision procedure (LEEP) was performed and the non-invasive therapeutic treatment was stopped for this patient. In addition, clinical observation plus relevant auxiliary examination has shown that, for all participants, there was no significant side effect during both the treatment and all follow-ups in the trial. Summarily, after one to three courses of REBACIN® treatment, 16 patients (88.89%) displayed both regression of hrHPV-associated cervical intraepithelial neoplasia from CIN2 to normal cervical cytology and clearance of high-risk HPV infection (Table 1).

**Table 1:** REBACIN® effect on the regression of hrHPV-associated CIN2.

**Case**	**HPV^**+**^ subtype**	**TCT**	**Pathology**	**p16**	**After 1**^****st****^ **course**			**After 2**^****nd****^ **course**			**After 3**^****rd****^ **course**	
					**HPV**	**TCT**	**Pathology**	**HPV**	**TCT**	**Pathology**	**HPV**	**TCT**
1	18	LSIL	CIN2	+	–	Normal	
2	16	ASCUS	CIN2	+	–	Normal	
3	16, 58,39	ASCUS	CIN2	+	58	Normal	CIN1	-	Normal	
4	52	LSIL	CIN2	+	52	Normal	CIN1	-	Normal	
5	59	HSIL	CIN2	+	–	Normal		
6	58	ASCUS	CIN2	+	58	Normal	Chronic inflammation	
7	58	LSIL	CIN2	+	–	Normal	
8	35, 52	LSIL	CIN2	+	–	Normal	
9	16	ASCUS	CIN2	+	–	Normal	
10	52	NILM	CIN2	+	–	Normal	
11	51, 56,68	LSIL	CIN2	+	–	Norma	
12	33, 52	ASCUS	CIN2	+	–	Normal	
13	16,18,39	LSIL	CIN2	+	16,18	LSIL	CIN2*	
14	16	ASCUS	CIN2	+	–	Normal	
15	16	ASCUS	CIN2	+	–	Normal	
16	16	ASCUS	CIN2	+	–	Normal	
17	68	NILM	CIN2	+	–	Normal	
18	16, 58	LSIL	CIN2	+	58	Normal	CIN1	16,58	Normal	CIN1	-	Normal

p16 immunostaining is recommended to clarify a final diagnosis in pathologic diagnosis of CIN2. If a CIN2 specimen is p16-positive, it will be classified as HSIL otherwise as LSIL. “+”represents “positive,” “–” represents “negative”; and *Transfer to the loop electrosurgical excision procedure (LEEP) surgery after the first course treatment.

Among the participants in this clinical treatment, 14 of 18 patients had fertility requirements. After one to three courses of REBACIN® treatment, all of them displayed both regression from HSIL and clearance of high-risk HPV infection. Three of them already had normal pregnancy and full-term vaginal delivery: the first patient (1^#^ patient, Table 1) delivered twice successfully and was followed up for 3 years without any recurrence of squamous intraepithelial lesion and high-risk HPV infection. The second patient (2^#^ patient, Table 1) delivered once successfully and was followed up for 1 year without any recurrence. However, 4 months after delivery, the ninth patient (9^#^ patient, Table 1) showed HPV16 positive which was the same type as her original infection, and was diagnosed as ASC-H (Atypical squamous cells, cannot exclude HSIL) with cervical cytology, and then was further diagnosed as CIN2 with colposcopy and cervical biopsy pathology, and also as diffuse positive expression of p16 with immunohistochemistry. The patient rejected surgical therapy again and accepted non-invasive therapeutic treatment of REBACIN®. However, after regression again, this patient was lost for further follow-up.

The patients were ultimately satisfied with the non-invasive intervention of REBACIN®. In this study, except for two of them who gave up for additional treatment, all 16 HSIL-patients showed both the regression from CIN2 to normal cervical cytology and clearance of high-risk HPV infection. Moreover, three of them already had normal pregnancy and full-term vaginal delivery successfully.

## Aptima HPV E6/E7 mRNA Assay

**Figure 3:**
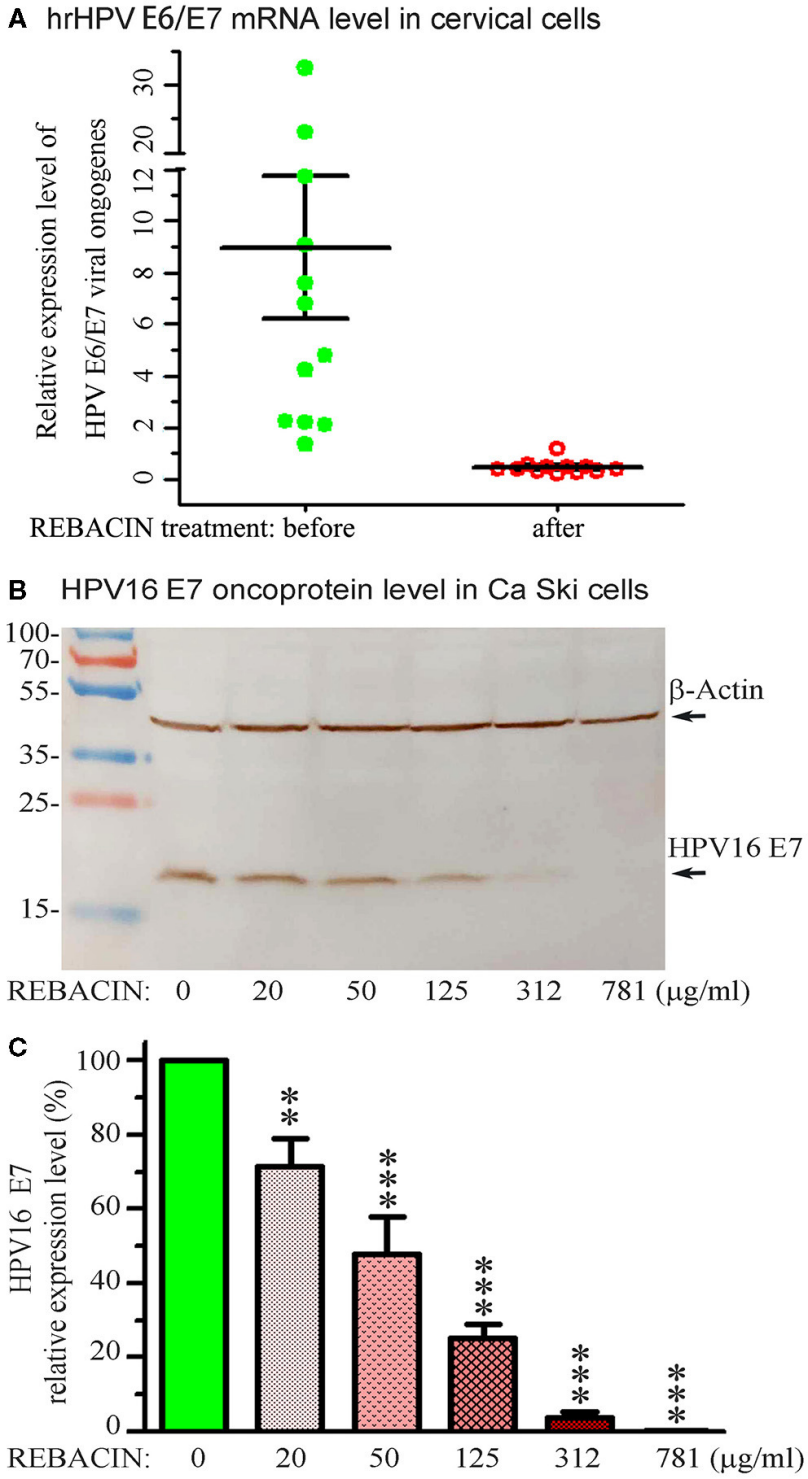
REBACIN® inhibits the expression of hrHPV E6/E7. **(A)** REBACIN® inhibits hrHPV E6/E7 mRNA. After one course (3 months) of REBACIN® treatment, the expression level of E6/E7 mRNAs in cervical specimens was evaluated using Aptima HPV E6/E7 mRNA assay. Specimens with S/CO values of ≥0.5 were considered as positive. **(B)** REBACIN® inhibits HPV16 E7 oncoprotein expression. Ca Ski cells with expression of HPV16 E7 were treated by different concentration of REBACIN® for 48 h, expression of the E7 oncoprotein was then evaluated by Western blot. REBACIN® clearly inhibited HPV16 E7 expression in a dose-dependent manner. **(C)** The results in **(B)** were quantified and shown in **(C)**. Histograms show mean ± SEM of three independent experiments performed in duplicate. **, *p* < 0.01; ***, *p* < 0.001 vs. control (0 μg/ml REBACIN®) analyzed by one-way analysis of variance followed by Bonferroni test (GraphPad Prism 6).

To determine whether REBACIN® treatment inhibits the transcription of high-risk HPV, a total of 12 patients with sustained infections of high-risk HPV, but without a high level of cervical lesions were enrolled in this investigation, age ranging from 22 to 57 years, with an average age of 39.5 years. After one course of treatment with REBACIN® as described above, right before the investigation and after follow-ups, HPV E6/E7 mRNA level of all patients was detected with Aptima HPV assay (Gen-Probe Inc., San Diego, CA) according to the manufacturing introduction. Assay results are interpreted on the basis of the signal-to-cutoff ratio (S/CO), and specimens with S/CO values of ≥0.5 were considered positive. As shown in Figure 3A, after one course (3 months) of REBACIN® treatment, the E6/E7 mRNA in hrHPV-infected patients was reduced remarkably, which most of them were became negative. Only in one patient, her E6/E7 mRNA level was above the control level but still obviously below than that without the treatment.

## SDS-Page and Western Blotting

Ca Ski (ATCC® CRL1550™), a human epidermoid cervical cancer cell line with endogenous expression of HPV-16/18, were purchased from Shanghai Enzyme Research Biotechnology Co., Ltd. The cells were cultured in RPMI 1,640 media containing 10% Fetal Bovine Serum, 100 units/ml of penicillin and 100 μg/ml of streptomycin, and incubated at 37°C in an atmosphere of 5% CO_2_. When the cells were grown near to 100% confluence, the culture media were replaced with the same fresh media, but containing different non-toxic concentration of REBACIN® (0-781 μg/ml). Following additional incubation of 48 h, total proteins in the cells were extracted with RIPA Lysis and Extraction Buffer containing 1x Halt™ Protease Inhibitor Cocktail an 5 mM EDTA Solution, separated by 10% regular SDS-PAGE, and transferred to nitrocellulose membrane. The oncoprotein E7 and β-Actin were detected with primary monoclonal antibodies (anti-HPV16 E7 and anti-β-Actin, Santa Cruz Biotechnology, Inc). REBACIN® significantly inhibited the expression of HPV16 E7 oncoprotein in a human epidermoid cervical cancer cell line of Ca Ski, and showed a dose-effect relationship (Figures 3B,C). The inhibitive effect could be detected as low to 20 μg/ml of REBACIN®, and the expression inhibition rate of HPV16 E7 oncoprotein could be up to 99%.

## Discussion

Standardized cervical cancer screening and the treatment for abnormal screening are the secondary prevention strategy to effectively block the progress of precancerous cervical lesions (7). According to the guidelines of 2012 ASCCP Colposcopy Standards on the indication of referral for colposcopy and the principle of the treatment of cervical high-grade lesions, HSIL-patients were routinely required for a surgical treatment, except in pregnant women and young women (9). Some HSIL-patients may refuse routine surgical treatment due to their personal concern. Furthermore, the surgical treatment can not clear the high-risk HPV infection, and thus the HSIL recurrence is still possible. Therefore, non-invasive clinical treatment of HSIL remains very imperative and urgently needed. In fact, this non-invasive therapy is also the consensus and development direction of cervical precancerous lesion's intervention.

The aim of this brief clinical trial was to demonstrate whether REBACIN®, a novel invasive intervention for high-risk HPV persistent infection,could have an effect for histopathologic regression of HSIL. 18 volunteers tested for HSIL (CIN2) were successfully enrolled, which were all in high-risk HPV infection. Their histopathological diagnosis of cervical biopsy specimen was all CIN2, which were further classified as HSIL according to p16 immunostaining. The most of these HSIL (CIN2)-patients enrolled in this study worried about the impact of cervical surgery on future fertility, while the other patients refused the surgery due to other concerns, so they all required non-invasive conservative treatment. After one to three courses of REBACIN® treatment, 88.89% (16/18) patients were shown on both regression of HSIL (CIN2) and clearance of high-risk HPV infection, including 72.22% (13/18) patients during first course of 3 months. The patients without complete regression after the first course treatment subsequently followed the same treatment for additional one or two courses, and also successfully regressed. 20-25% of spontaneous regression rate during 6 months was reported in CIN2/3-patients (11), and around 50% of spontaneous regression rate during 2 year (12) and 16 months (13) was also reported for CIN2-patients. Factors, such as age, HPV subtype, LSIL/ASCUS at initial cytology, and also follow-up time, influence the spontaneous regression of CIN2 (14–18). CIN2-patients with HPV16 infection had much lower spontaneous regression rate than the others (16, 17). In current study, 88% (7/8) of HSIL (CIN2) patients with HPV16 infection displayed not only the regression, but also clearance of virus infection. Therefore, REBACIN® intervention is indeed a potential non-invasive therapy for HSIL (CIN2) regression. However, due to the limitation of participants in this observation, more data from prospective studies are needed to accurately evaluate the REBACIN® effects on HSIL regression. Meanwhile, for the participants of poor regression, we observed recurrent fungal vaginitis and bacterial vaginosis during the REBACIN® treatment. This indicated that recurrent vaginal infections and abnormal vaginal flora may be affecting factors for HSIL regression. This is also consistent with that vaginal microecological abnormalities are associated with persistent HPV infection and cervical precancerous lesions (19–21).

HPV E6 and E7 oncoproteins can trick the cells to become oncogenic in the process of viral replication. The E6 and E7 are therefore the major oncoplayers driving the process of HPV-mediated cervical tumorigenesis. Thus, the E6 and E7 represent the most effective targets for therapeutics as it can ensure the eradication of all cervical cancer cells by bringing down any or all of the cancer hallmarks, and overexpression of E6 and E7 in HPV-infected malignant cells plays a key role in HSIL (22–25). In line with these studies, in a severe combined immunodeficiency (SCID) mouse model, REBACIN® was previously reported to suppress the growth of tumors induced by expression of E6 and E7 oncogenes of either HPV16 or HPV18 (10). In addition, targeting in the oncoprotein of HPV E6 and E7, therapeutic vaccine of high-grade cervical intraepithelial neoplasia has been developed as an alternative to surgery, to preserve future reproductive outcomes (11, 26, 27). In this study, REBACIN® treatment did notably suppress the expression of E7 oncoprotein with the relations of dose-effect in the absence of cellular toxicity. Thus, the obvious effects of REBACIN® on the regression of high-grade squamous intraepithelial lesion probably due to the suppression of E6 and E7 oncoproteins, which a mechanism is worth further investigation.

## Patient Perspective

The first patient (1# patient, Table 1): “In a routine examination, I tested positive for high-risk HPV 18 in Tianjin First Central Hospital, and they admitted me. After TCT and HPV DNA double test, I was transferred for a colposcopic observation and then cervical biopsy pathology. Finally, I was diagnosed as HSIL-patient with CIN2 and positive of diffuse p16. My doctor told me that I need to do a treatment of surgery. However, I was so worry about the adverse effect of surgery, since I really want children. I told my doctor if she had any other options? According to my request, the doctor recommended me to try a new biological product, REBACIN®. She also told me about this newly created non-invasive treatment in details and I accepted a one-course (3 months) treatment. The doctor and nurses were excellent, and they communicated with me very often. Two months after the treatment, I was diagnosed as normal TCT and negative of high-risk HPV. I was so lucky to deliver twice successfully and don't get any recurrence of squamous intraepithelial lesion and high-risk HPV infection during all follow-ups in 3 years. I am very grateful to my doctor and the innovative non-invasive treatment of REBACIN®.”

## Conclusion

The cases reported here are certainly encouraging, except for two patients who gave up during the treatment, all the others of 16 patients showed both the regression of CIN2 to normal cervical cytology and clearance of hrHPV infection. Moreover, so far, three of them have already had normal pregnancy and successful full-term vaginal delivery. It is the first time to report a non-invasive strategy in HSIL treatment, which creates a new clinical approach and option in addition to standard invasive treatment of surgery for HSIL patients, especially for the patients with fertility requirements. Due to the great compliance of non-invasive intervention with REBACIN®, patients can use it by themselves under the guidance of doctors without hospitalization, which is of great practical significance for the clinical application, especially for early diagnosis and treatment (even before the stage of HSIL), which is worthy of further research, verification and application to prevent cervical cancer.

## Data Availability Statement

The original contributions presented in the study are included in the article/Supplementary Material, further inquiries can be directed to the corresponding author/s.

## Ethics Statement

The studies involving human participants were reviewed and approved by the ethics committee of Tianjin First Central Hospital. The patients/participants provided their written informed consent to participate in this study.

## Author Contributions

FW, RL, and YM: investigation, data collection, and writing—review and editing. DFW: supervision, methodology, formal analysis, writing—original draft, and writing—review and editing. LHD and SW: resources and writing—review and editing. GYW: data collection, formal analysis, and writing—review and editing. CFZ and QXQ: conceptualization, resources, supervision, investigation, formal analysis, writing—original draft, and writing—review and editing. All authors have read and agree to the published version of the manuscript.

## Conflict of Interest

The authors declare that the research was conducted in the absence of any commercial or financial relationships that could be construed as a potential conflict of interest.
