# Supplementary material for: Case Report: Noninvasive Clinical Intervention of REBACIN® on Histologic Regression of High Grade Cervical Intraepithelial Neoplasia
Source: Front Med (Lausanne). 2021 Jul 20;8:627355. doi: 10.3389/fmed.2021.627355 (PMC8329360; doi:10.3389/fmed.2021.627355)
Supplement: Supplementary file 1 [file Data_Sheet_1.PDF]

Supplementary Material

Figure S1.

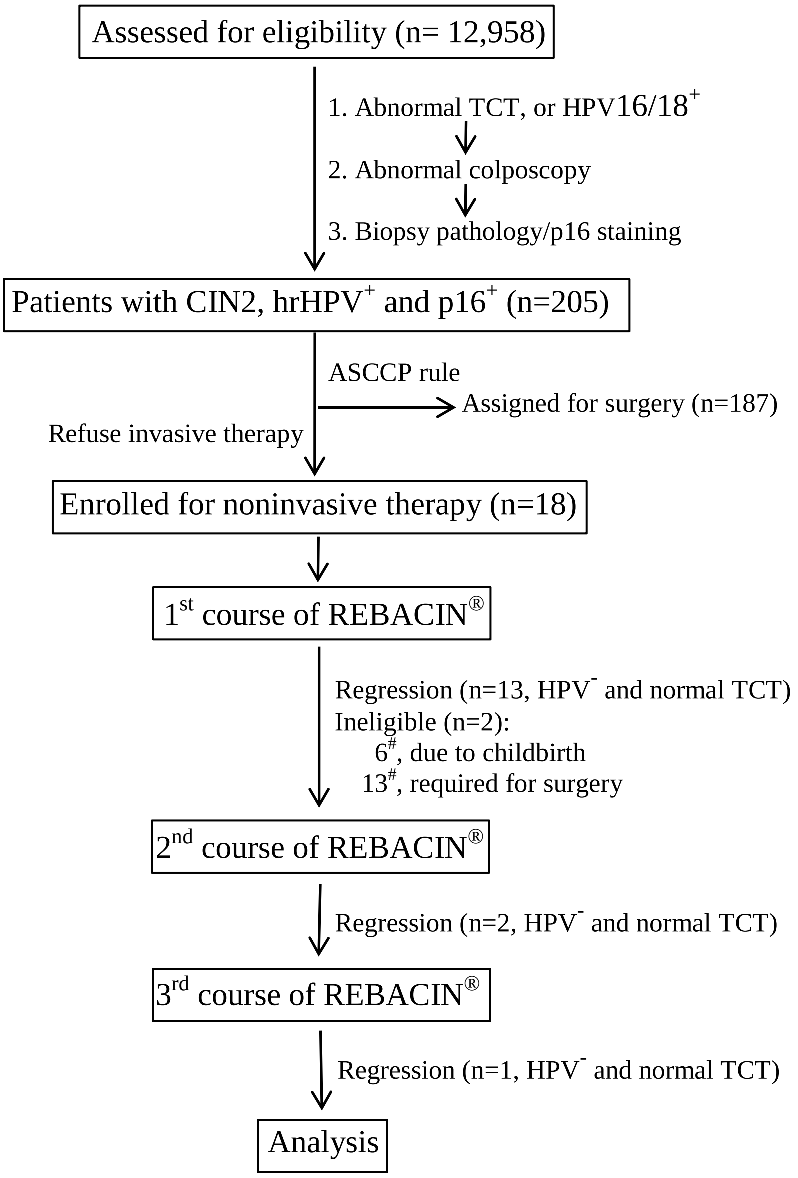

Figure S1. Consort flow diagram. 12,958 patients were assessed for eligibility. Patients with abnormal TCT or HPV16/18^{+} were further administered for colposcopy, following the histopathology and p16 staining if abnormal symptom was observed under colposcopy. Note that: HPV16/18^{+} represents positive HPV16, 18 or both, and p16^{+} represents positive staining of p16 protein.

Table S1. **Characteristics of participants enrolled in this clinical treatment.**

Case	Age	G/P	HPV^+ subtype	TCT	Colposcopic evaluation	Cervical histopathology	p16 immuno-staining
1	25	0/0	18	LSIL	HSIL	CIN2	Positive
2	25	0/0	16	ASCUS	LSIL	CIN2	Positive
3	27	0/0	16,58,39	ASCUS	HSIL	CIN2	Positive
4	26	0/0	52	LSIL	LSIL	CIN2	Positive
5	35	0/0	59	HSIL	HSIL	CIN2	Positive
6	24	0/0	58	ASCUS	LSIL	CIN2	Positive
7	31	1/1	58	LSIL	LSIL	CIN2	Positive
8	50	1/1	35,52	LSIL	LSIL	CIN2	Positive
9	30	0/0	16	ASCUS	LSIL	CIN2	Positive
10	24	1/1	52	NILM	LSIL	CIN2	Positive
11	30	0/0	51,56,68	LSIL	LSIL	CIN2	Positive
12	26	1/0	33,52	ASCUS	LSIL	CIN2	Positive
13	23	0/0	16,18,39	LSIL	LSIL	CIN2	Positive
14	24	0/0	16	ASCUS	LSIL	CIN2	Positive
15	28	1/1	16	ASCUS	LSIL	CIN2	Positive
16	27	0/0	16	ASCUS	HSIL	CIN2	Positive
17	44	7/2	68	NILM	LSIL	CIN2	Positive
18	38	4/1	16,58	LSIL	LSIL	CIN2	Positive

Note that: HPV^+=HPV positive, G/P=Gestation/Parturition, TCT=Thinprep Cytologic Test, HPV=human papillomavirus, CIN2=cervical intraepithelial neoplasia grade 2, HSIL=high-grade squamous intraepithelial lesions, LSIL=low-grade squamous intraepithelial lesions, ASCUS=atypical squamous cells ofundetermined significance, NILM= negative for intra-epithelial lesions and malignancy.
